# Supplementary material for: Clinical Study of the Relationship between Sjögren Syndrome and T-Cell Large Granular Lymphocytic Leukemia: Single-Center Experience
Source: Int J Mol Sci. 2022 Nov 1;23(21):13345. doi: 10.3390/ijms232113345 (PMC9656665; doi:10.3390/ijms232113345)
Supplement: Supplementary file 1 [file ijms-23-13345-s001.zip › ijms-1912475-supplementary.pdf]

Table S1. *STAT3* mutations in the cohort of 45 patients with T-LGL leukemia and rheumatic disease but without Sjögren syndrome

Patient no. / Sex	Diagnosis	Samples for *STAT3* testing	Variants of *STAT3* mutation
1. / F	RA	PB	–
2. / F	RA	PB	Y640F; S614R
3. / F	RA	PB	S614R
4. / F	RA	PB	S614R
5. / M	RA	PB	Y640F
6. / F	RA	PB<br>BM	Y640F<br>Y640F
7. / F	RA	PB	N647I
8. / M	RA	PB	Y657_K658insY
9. / M	RA	PB	N647I
10. / F	RA	PB	S614R
11. / M	RA	PB<br>BM	Y657_K658insY<br>Y657_K658insY
12. / M	RA	PB	S614R; G618R
13. / F	RA	PB	–
14. / M	RA	PB	–
15. / F	RA	PB	–
16. / F	RA	PB<br>BM	–<br>Y640F
17. / F	RA	PB	–
18. / M	RA	PB	–
19. /F	RA	PB<br>BM	–<br>–
20. / M	RA	PB<br>BM	N647I<br>N647I
21. / F	RA	PB	Y640F
22. / F	RA	PB<br>BM	–<br>–
23. / F	RA	PB	–
24. / F /	RA	PB<br>BM	–<br>–
25. / M	RA	PB	D661Y
26. / F	RA	PB	–
27. / M	RA	PB<br>BM	Y640F; D661Y<br>Y640F; D661Y
28. / F	RA	PB<br>BM	Y640F<br>Y640F
29. / F	RA	PB	–
30. / F	RA	PB	–
31. / F	RA	Spleen	–
32. / M	RA	PB<br>BM<br>Spleen	–<br>Y640F<br>Y640F
33. / M	RA	Spleen	S614R; N647I; Y640F
34. / F	RA	PB<br>BM<br>Spleen	–<br>S614R<br>S614R

35. / F	RA	PB<br>Spleen	D661Y<br>D661Y
36. / F	RA	PB	−
37. / M	RA	PB	Y640F; D661V; S614R
38. / F	RA	PB	G618R
39. / F	SLE	PB	Y640F
40. / F	SLE	PB	K658R
41. / F	Ankylosing spondylitis	PB	D661Y
42. / M	IIM	PB	−
43. / F	IIM	PB	−
44. / F	IIM	PB	−
45. / F	Primary antiphospholipid syndrome	PB	D661Y

RA, rheumatoid arthritis; SLE, systemic lupus erythematosus; IIM, idiopathic inflammatory myopathy; *STAT3*, signal transducer and activator of transcription 3 gene; BM, bone marrow; PB, peripheral blood; −, negative/absent
